# Comparison of the Efficacy of ShuoTong Ureteroscopy and Simple Flexible Ureteroscopy in the Treatment of Unilateral Upper Ureteral Calculi

**DOI:** 10.3389/fsurg.2021.707022

**Published:** 2021-09-27

**Authors:** Longhui Lai, Wenzhao Zhang, Fangjian Zheng, Tao Wang, Peide Bai, Zhengsheng Liu, Jiaxin Zheng, Zhiqiang Shao, Bo Duan, Huiqiang Wang, Jinchun Xing, Huixin Chen, Yushan Huang, Bin Chen

**Affiliations:** ^1^The Key Laboratory of Urinary Tract Tumors and Calculi, Department of Urology Surgery, The First Affiliated Hospital, School of Medicine, Xiamen University, Xiamen, China; ^2^The School of Clinical Medicine, Fujian Medical University, Fuzhou, China; ^3^Xiamen University Laboratory Animal Center, Xiamen University, Xiamen, China; ^4^Department of Urology Surgery, Zhangzhou Hospital of Traditional Chinese Medicine, Zhangzhou, China; ^5^Department of Urology Surgery, Anxi County Hospital of Traditional Chinese Medicine, Quanzhou, China

**Keywords:** unilateral upper ureteral calculi, ShuoTong ureteroscopy, flexible ureteroscopy, efficacy and safety, stone-free rate

## Abstract

**Background:** ShuoTong ureteroscopy (Sotn-ureteroscopy, ST-URS), a new lithotripsy operation method developed on the basis of ureteroscopy, is widely used to treat ureteral stones in China. Its composition includes rigid ureteral access sheath, standard mirror, lithotripsy mirror, and ShuoTong perfusion aspirator (ST-APM). Here, we compared the efficacy and safety of the ST-URS and the flexible ureteroscope (F-URS) holmium laser lithotripsy in the treatment of unilateral upper ureteral calculi.

**Methods:** Retrospective analysis was conducted on the clinical data of 280 patients who met the inclusion 1) urinary tract CT was diagnosed with unilateral single upper ureteral calculi above the L4 lumbar spine; 2) patient age was from 18 to 80 years old; 3) patients were informed and consented to this study; and 4) patients were approved by the hospital ethics committee (proof number: KY-2019-020) and the exclusion criteria for unilateral upper ureteral calculi in the First Affiliated Hospital of Xiamen University from January 2018 to November 2020, and they were divided into the ST-URS group and the flexible ureteroscopy (F-URS) group.

**Results:** The stone-free rate of 1 day after operation of the ST-URS group was significantly higher than the F-URS group (63.71 vs. 34.62%, *P* < 0.0001). The operative time (38.45 vs. 46.18 min, *P* = 0.005) and hospitalization cost (27,203 vs. 33,220 Yuan, *P* < 0.0001) of the ST-URS group were significantly lower than the F-URS group. There were no significant differences in the success rate of ureteral access sheath placement, operative blood loss, stone-free rate of 1 month after operation, postoperative complications, postoperative hospital stay, and postoperative visual analog scale (VAS) pain score between the two groups (*P* > 0.05). In subgroups of a diameter of calculi ≥ 1.5 cm, calculi CT numerical value ≥ 1,000 Hounsfield unit and the preoperative hydronephrosis range ≥ 3.0 cm, ST-URS shows more advantages in the operative time, stone-free rate of 1 day after the operation, the hospitalization cost, and the incidence of postoperative complications.

**Conclusion:** In unilateral upper ureteral stones treated with a holmium laser, compared with the simple F-URS, the ST-URS has a shorter operative time, lower hospitalization cost, and a higher stone-free rate of 1 day after the operation, suggesting that the ST-URS could be more widely applied in clinics.

## Introduction

Urolithiasis is a common disease in urology with an incidence rate of 1–5%, and the recurrence rate in 10 years can reach 50% (1, 2). Recently, the incidence rate of urolithiasis has been increasing year by year (3). Upper ureteral calculus is one of the main types of urinary calculi and its main clinical characteristics are renal colic and hematuria. While the diameter of a stone is more than 1 cm, it can easily cause obvious urinary tract obstruction, resulting in kidney function damage in a short time and seriously affecting the clinical prognosis (4). Clinically, the treatment of upper urinary tract stones depends on the surgery. Hospitalization and postoperative morbidity of the traditional open surgery are significantly higher than shock wave lithotripsy and endourological procedures (5). Currently, indications for traditional open stone surgery are rare, so it is less used in clinical practice (6). At present, the common methods for clinical treatment of upper ureteral calculi include extracorporeal shock wave lithotripsy, rigid ureteroscopy, flexible ureteroscopy (F-URS), and percutaneous nephrolithotripsy (PCNL) (7). The treatment of ureteral calculi with the ureteroscopy has many advantages, such as smaller trauma, quick recovery, and high stone clearance rate, and is considered as the first choice for the treatment of upper ureteral calculi by many authors (8–11). Negative pressure combined with ureteroscopy, also called ShuoTong ureteroscopy (Sotn-ureteroscopy, ST-URS), is a new type of stone removal surgery in China in recent years. ST-URS can suck out larger stones while crushing the stones, reduce the residual stone fragments and the residual stone rate, and the risk of postoperative stone-street formation. In addition, ST-URS can maintain the renal pelvis at low pressure by its vacuum suction to reduce the risk of infection and bleeding caused by prolonged surgery. Importantly, the flexible ureteroscope can be inserted through a rigid ureteral access sheath (UAS) to treat kidney stones and residual stones, thereby increasing the stone-free rate (SFR). In this study, the clinical application effects of two surgical methods for treating unilateral upper ureteral calculi were compared between ST-URS and simple F-URS and these findings provide a theoretical basis for the clinical treatment of upper ureteral calculi.

## Materials and Methods

### Clinical Information

Retrospectively, analysis was conducted on the clinical data of patients who were diagnosed with unilateral upper ureteral calculi and treated with ST-URS and F-URS in the First Affiliated Hospital of Xiamen University from January 2018 to November 2020. The inclusion and exclusion criteria were listed as shown in Table 1. Inclusion criteria: 1) urinary tract CT [noncontrast computed tomography (NCCT)] was diagnosed with unilateral single upper ureteral calculi above the L4 lumbar spine; 2) patient age was from 18 to 80 years old; 3) patients were informed and consented to this study; and 4) patients were approved with the hospital ethics committee (proof number: KY-2019-020). Exclusion criteria: 1) patients were with complex kidney stones, bladder stones, renal tuberculosis, renal tumors, renal dysfunction, acute or chronic nephritis, and nephrotic syndrome; 2) patients were with severe urethral stricture and other urinary malformation; 3) patients have cardiopulmonary dysfunction and cannot tolerate surgical treatment; 4) patients have abnormal coagulation function; 5) patients were with a positive culture of urine bacteria; and 6) the preoperative urine white blood cell count was more than 500/μl. A total of 280 patients were enrolled according to the aforementioned criteria. According to the surgical methods, the patients were divided into the ST-URS group (124 cases) and the F-URS group (156 cases).

**Table 1:** Inclusion criteria and Exclusion criteria.

**Inclusion criteria**	**Exclusion criteria**
Urinary tract CT (non-contrast Computed tomography) was diagnosed with unilateral single upper ureteral calculi above the L4 lumbar spine	Patients were with complex kidney stones, bladder stones, renal tuberculosis, renal tumors, renal dysfunction, acute or chronic nephritis and nephrotic syndrome
Patient age was from 18 to 80 years old	Patients were with severe ureteral stricture and other urinary malformation
Patients were informed and consented to this study	Patients have cardiopulmonary dysfunction and cannot tolerate surgical treatment
Patients were approved with the hospital ethics committee (proof number: KY-2019-020)	Patients have abnormal coagulation function
	Patients were with positive culture of urine bacteria
	The preoperative urine white blood cell count was more than 500/μL

### Main Surgical Instruments and Materials

The following instruments were used in this study: a URF-P5 flexible ureteroscope (Olympus, Tokyo, Japan), flexible laser (200 pm, holmium laser fiber, Lumenis, Beijing, China), a 0.035-foot nickel-titanium super smooth guide wire (0.888 mm × 150 cm, C.R. Bard Inc., Murray Hill, NJ, USA), a 1.7-Fr basket catheter (Zero tipped, Boston Scientific Corp, Natick, MA, USA), an 8.5/9.8 rigid ureteroscope and ST-URS (Jiangmen, China), namely, a standard ureteroscope (F7.5/11.5), a gravel ureteroscope (F4.5/6.5), a rigid ureteral channel sheath (F11.5/13.5), and a ShuoTong perfusion aspirator (ST-APM).

### Surgical Procedure

For the ST-URS group, after general anesthesia, the patients were placed in the lithotomy position while and the device was connected. A standard mirror (F7.5/11.5) was combined with a rigid UAS (F11.5/13.5), and the F11.5/13.5 rigid UAS was inserted into the urethra under direct vision under the guidance of a super smooth guidewire. Then, the UAS was inserted into the interureteric ridge and was fixed at the position where the ureteral calculus is located on the affected side. Subsequently, the rigid UAS was left in place, and the ShuoTong standard mirror was removed. Next, a special vacuum suction device was connected to the end of the rigid UAS, which was connected with the mastering perfusion aspirator so that the collection system and negative pressure system formed a closed loop, thus establishing a working channel. Then, the gravel mirror is placed in the rigid UAS with a perfusion aspirator, and a 200 μm holmium laser fiber was placed in the operation channel of the gravel mirror. A holmium laser with a power of 8–30 W (0.4–1.0 J/20~30 Hz) was used to crush the stone into pieces or powder. In the gravel process, the interspace between the shaft of the gravel mirror and the rigid UAS allowed continuous outflow by vacuum suctioning, and stone fragments flow out from this interspace. The operator can regulate the negative pressure of the suctioning system through the negative pressure adjustment button at the end of the rigid UAS. If the stone moved up to the lower calyx during surgery, exit the gravel mirror, and the flexible ureteroscope was placed into the outer sheath. Stones in the lower calyx were moved into the renal pelvis or upper calyx by using a 1.7-Fr basket catheter and the flexible ureteroscope was replace with a gravel mirror to clear stones. After the ureter and the renal pelvis were viewed and no obvious stone fragments were observed, perfusion and vacuum suction was stopped. The gravel mirror was then removed, and a standard mirror was put in its place and was fastened to the rigid outer sheath. The standard mirror and the rigid outer sheath are exited simultaneously under visual vision. The renal pelvis region and ureteral mucosal damage were observed when the standard mirror was removed. Then, an F7 D-J tube was inserted, and an F20 three-chamber catheter was inserted. We also made a video to show the surgical procedure of ST-URS as shown on the website of *Frontiers in Surgery*. For the F-URS group, the operation was performed as described previously (12).

### Observation Index

It includes operative time, operative blood loss, SFR of 1 day after the operation, SFR of 1 month after the operation, the incidence of postoperative complications, the success rate of UAS placement, creatinine level, hospitalization cost, postoperative hospital stay, postoperative catheter extraction time, and postoperative visual analog scale (VAS) pain score.

### Judgment Standard

Complications were classified according to the modified Clavien classification system and the infectious complications were classified according to the standardized classification system of Francesco Berardinelli (13, 14). The occurrence of fever postoperatively was defined as an increase in the body temperature to > 38°C, which persisted for 48 h (15). The stone size was measured based on the maximal diameter of the stone by three-dimensional reconstruction NCCT is used as the size of the stone. The SFR was defined as the presence of no stones or only residual stone fragments of <4 mm in diameter (16–18). The CT scan was re-examined 1 month after the operation and there were no residual stones or clinically meaningless stones suggesting the operation was successful. The hospitalization cost was calculated with the sum of all examinations, medicines, surgical consumables, and surgical operation expenses during the hospitalization period.

### Statistical Analysis

SPSS 23.0 software (IBM SPSS; Armonk, NY, USA) was used for the statistical analysis. Measurement data are presented as the means ± SD, Student's *t*-test was applied to continuous data with normal distribution, and the Mann–Whitney rank-sum test was applied to continuous data with the nonnormal distribution. For data presented as percentages (%), the χ^2^-test was applied for group comparisons. *P* < 0.05 was considered to indicate a statistically significant difference.

## Results

### Compositions and Surgical Procedures of ST-URS

**Figure 1:**
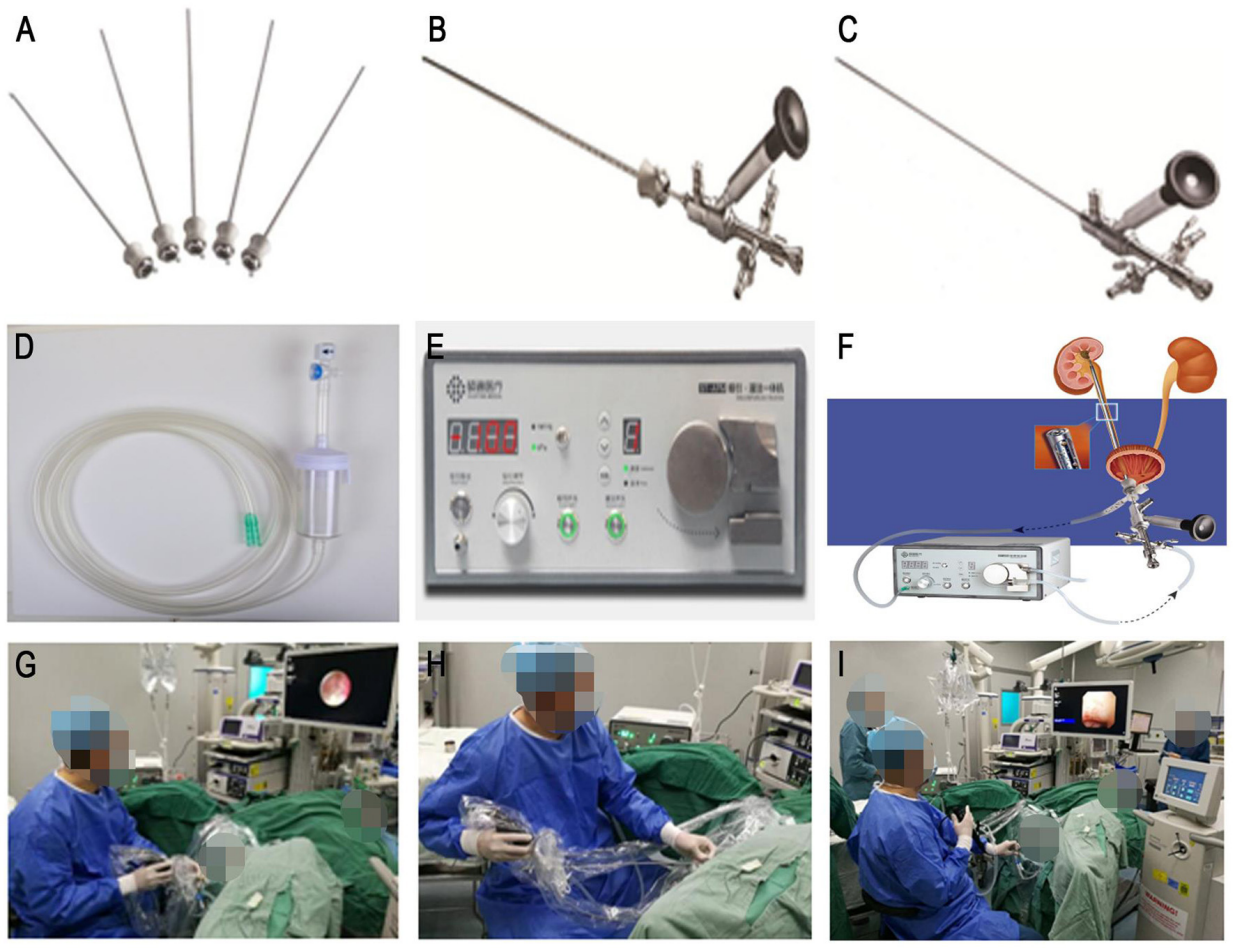
ShuoTong mirror compositions and surgical procedures. **(A)** The mirror sheath portion of the standard mirror. **(B)** Standard mirror. **(C)** Gravel mirror. **(D)** Adjustable negative pressure suction device and stone collector. **(E)** Vacuum suction system, perfusion system. **(F)** Connection diagram. **(G)** Use of the gravel mirror. **(H)** Use of vacuum suction to remove stone fragments and powder. **(I)** The rigid outer sheath is inserted into the flexible ureteroscope for examination.

ShuoTong ureteroscopy is a new lithotripsy operation method developed on the basis of ureteroscopy in China in recent years. It is a system that combines lithotripsy and stone removal. Its composition includes rigid UAS, standard mirror, lithotripsy mirror, and ST-APM. Compared with the F-URS, the biggest characteristic and advantage of the ST-URS is the negative pressure perfusion aspirator (Figure 1).

### Efficacy and Safety Analysis of ST-URS and F-URS

In the ST-URS group, there were 82 men and 42 women, their age was from 24 to 79 (average: 49.4 ± 12.8) years old, the average diameter of ureteral stones was 1.37 ± 0.49 cm, the CT numerical value of calculus was 1,003.1 ± 332.7 Hounsfield unit (HU), and the preoperative hydronephrosis range was 2.9 ± 1.2 cm. In the F-URS group, there were 104 men and 52 women, their age was from 22 to 79 (average: 49.7 ± 13.2) years old, the average diameter of ureteral stones was 1.35 ± 0.43 cm, the stone CT numerical value was 1,055.5 ± 341.6 HU, and the preoperative hydronephrosis range was 2.7 ± 1.1 cm. There was no significant difference between the two groups of patients in general information such as age, body mass index, preoperative white blood cell count, preoperative blood neutrophil ratio, the diameter of calculus, the CT numerical value of calculus, and preoperative hydronephrosis range statistically (*P* > 0.05, Table 2).

**Table 2:** Comparison of the basic information and Surgical effect in the two groups.

**Variables**	**Total**	**ST-URS**	**F-URS**	***P***
Cases	280	124	156	-
Sex (M/F)	186/94	82/42	104/52	-
Age (years)	49.6 ± 13.0	49.4 ± 12.8	49.7 ± 13.2	0.886^t^
BMI	24.3 ± 3.3	24.5 ± 3.0	24.0 ± 3.6	0.206^t^
Stone size (cm)	1.36 ± 0.46	1.37 ± 0.49	1.35 ± 0.43	0.946^u^
Stone CT numerical value (Hu)	1032.3 ± 338.1	1003.1 ± 332.7	1055.5 ± 341.6	0.189^u^
Hydronephrosis (cm)	2.8 ± 1.2	2.9 ± 1.2	2.7 ± 1.1	0.195^*u*^
Preoperative white blood cell count (× 10^9^/L)	6.72 ± 1.70	6.73 ± 1.52	6.72 ± 1.84	0.614^u^
Preoperative bloodneutrophil ratio (%)	58.6 ± 8.5	59.5 ± 8.2	57.8 ± 8.6	0.099^t^
Operative time (min)	42.76 ± 23.29	38.45 ± 21.09	46.18 ± 24.42	0.005^u^
Operative blood loss (ml)	4.24 ± 6.65	4.22 ± 7.86	4.26 ± 5.51	0.361^u^
Success rate ofUAS placement	97.14% (272/280)	97.58%(121/124)	96.79%(151/156)	0.975^χ^
SFR of 1 dayafter operation	47.50% (133/280)	63.71%(79/124)	34.62% (54/156)	< 0.0001^χ^
SFR of 1 monthafter operation	83.93% (235/280)	87.10%(108/124)	81.41%(127/156)	0.198^χ^
Hospitalization cost(Yuan)	30,556 ± 7,077	27,203 ± 7,134	33,220 ± 5,798	< 0.0001^t^
Postoperative hospital stay (day)	2.46 ± 1.08	2.49 ± 1.20	2.44 ± 0.98	0.939^u^
Postoperative catheter extraction time (day)	1.56 ± 0.81	1.49 ± 0.71	1.61 ± 0.83	0.138^u^
Postoperative VAS pain score	1.00 ± 0.33	0.98 ± 0.41	1.02 ± 0.24	0.300^t^
Creatinine before the operation (μmol/l)	80.7 ± 24.3	80.6 ± 20.6	80.8 ± 27.0	0.954^t^
Creatinine 1 day after the operation (μmol/l)	75.2 ± 21.7	74.3 ± 17.4	75.9 ± 24.5	0.546^t^
*P*	< 0.0001^t^	< 0.0001^t^	< 0.0001^t^	-

*ST-URS, negative pressure combined ureteroscopy (Sotn-ureteroscopy); F-URS, flexible ureteroscopy; BMI, Body Mass Index; UAS, ureteral access sheath; SFR, stone-free rate; VAS, visual analogue scale; P < 0.05 as statistically significant*;

t *Using the Student's t test*;

χ *Using the Chi-squared test*;

u *Using the Mann-Whitney U-test*.

The operation time of the ST-URS group was shorter (38.45 vs. 46.18 min, *P* = 0.005) and the SFR of 1 day after the operation was higher (63.71 vs. 34.62%, *P* < 0.0001) than that of the F-URS group as shown in Table 2. However, there were no statistically significant differences between the two groups in operative blood loss, SFR of 1 month after the operation, the incidence of postoperative complications, and the success rate of UAS placement (*P* > 0.05, Table 2). In addition, we analyzed the hospitalization cost of these two groups and found that the ST-URS group was significantly less than that of the F-URS group (*P* < 0.0001, Table 2). There were no statistically significant differences in the postoperative hospital stay, postoperative catheter removal time, and postoperative VAS pain score between the two groups (*P* > 0.05, Table 2).

In addition, we analyzed the creatinine level between these two groups and found that there was no statistical significance in the comparison of creatinine level before and 1 day after the surgery between the two groups (*P* > 0.05), while the creatinine level of 1 day after the surgery in these two groups is significantly lower than that of before the surgery (*P* < 0.0001, Table 2).

In the F-URS group, Clavien I complications were noted in six cases, namely, fever in three cases and hematuria in three cases. Clavien II complications were noted in 14 cases, namely, ureteral injury in 2 cases, urinary tract infection in 11 cases, and systemic inflammatory response syndrome (SIRS) in 1 case. Clavien IV complications were noted in one case with septic shock. In the ST-URS group, Clavien I complications were noted in three cases with fever. Clavien II complications were noted in six cases with urinary tract infection. No Clavien III–V complications were noted. The incidence of surgical complications of the ST-URS group was lower than the F-URS group (7.26 vs. 13.46%, *P* = 0.095, Table 3).

**Table 3:** Comparison of the Surgical complication in the two groups.

	**Total, N (280)**	**ST-URS, N (124)**	**F-URS, N (156)**	***P***
**Clavien Grade I**				
Hematuria	3(1.07%)	0	3(1.92%)	0.333^χ^
**Clavien Grade II**				
Ureteral injury	2(0.71%)	0	2(1.28%)	0.505^χ^
**Clavien Grade III**				
Urethral stricture	0	0	0	-
Infection	25(8.93%)	9(7.26%)	16(10.26%)	0.382^χ^
Fever (> 38°C) (G I)	6(2.14%)	3(2.42%)	3(1.92%)	1.000^χ^
Urinary tract infection (GII)	17(6.07%)	6(4.83%)	11(7.69%)	0.4410^χ^
SIRS/Sepsis (GII)	1(0.36%)	0	1	1.000^χ^
Septic shock (GIV)	1(0.36%)	0	1	1.000^χ^
Total	30(10.71%)	9(7.26%)	21(13.46%)	0.095^χ^

*P < 0.05 as statistically significant*;

χ *Using the Chi-squared test*.

### Subgroup Analysis of ST-URS and F-URS

Furthermore, we analyzed the operation time of these two groups and found when the diameter of calculi ≥ 1.5 cm, the operation time of the ST-URS group was shorter than that of the F-URS group (42.87 vs. 52.41 min, *P* = 0.01). The SFR of 1 day after the surgery was 51.06% in the ST-URS group and that was 20.41% in the F-URS group, and the difference was statistically significant (*P* = 0.002). The hospitalization cost analysis found ST-URS group was significantly less than that of the F-URS group (*P* < 0.0001). The incidence of surgical complications was 6.38% in the ST-URS group and that was 18.37% in the F-URS group (*P* = 0.076). There were no significant differences between these two groups in the operative blood loss and the SFR of 1 month after operation (*P* > 0.05, Table 4). When the calculi CT numerical value ≥ 1,000 HU, the operation time of the ST-URS group was shorter than that of the F-URS group (40.10 vs. 49.43 min, *P* = 0.01). The SFR of 1 day after the surgery was 60.66% in the ST-URS group and that was 25.29% in the F-URS group (*P* < 0.0001). The incidence of surgical complications (3.28%) in the ST-URS group was dramatically decreased than that of the F-URS group (13.79%, *P* = 0.031). The hospitalization cost analysis of these two groups found that the ST-URS group was significantly less than that of the F-URS group (*P* < 0.0001). However, there were no significant differences between the two groups in the operative blood loss and the SFR of 1 month after operation (*P* > 0.05, Table 5).

**Table 4:** Comparison of Surgical effect in the two groups while the diameter of calculi ≥ 1.5 cm.

**Variables**	**Total**	**ST-URS**	**F-URS**	***P***
Cases	96	47	49	-
Sex (M/F)	75/21	39/8	36/13	-
Age (years)	49.3 ± 13.3	48.6 ± 13.4	50.0 ± 13.4	0.593^t^
BMI	24.6 ± 3.2	24.8 ± 3.0	24.4 ± 3.3	0.603^t^
Stone size (cm)	1.87 ± 0.32	1.89 ± 0.32	1.85 ± 0.32	0.412^u^
Stone CT numerical value (Hu)	1108.8 ± 305.0	1113.7 ± 301.8	1104.1 ± 311.0	0.878^t^
Hydronephrosis (cm)	2.9 ± 1.3	3.0 ± 1.2	2.8 ± 1.3	0.418^t^
Operative time (min)	49.20 ± 20.62	42.87 ± 15.73	52.41 ± 19.49	0.010^t^
Operative blood loss (ml)	4.44 ± 6.71	4.85 ± 8.24	4.04 ± 4.87	0.904^u^
SFR of 1 dayafter operation	35.42% (34/96)	51.06%(24/47)	20.41% (10/49)	0.002^χ^
SFR of 1 monthafter operation	81.25% (78/96)	85.11%(40/47)	77.55% (38/49)	0.343^χ^
Hospitalization cost(yuan)	29,698 ± 5,560	26,842 ± 4,285	32,439 ± 5,285	< 0.0001^t^
Total complication rate	12.50%(12/96)	6.38%(3/47)	18.37%(9/49)	0.076^χ^

*ST-URS, negative pressure combined ureteroscopy (Sotn-ureteroscopy); F-URS, flexible ureteroscopy; BMI, Body Mass Index; SFR, stone-free rate; P < 0.05 as statistically significant*;

t *Using the Student's t test*;

χ *Using the Chi-squared test*;

u *Using the Mann-Whitney U-test*.

**Table 5:** Comparison of Surgical effect in the two groups while the calculi CT numerical value ≥ 1,000 Hu.

**Variables**	**Total**	**ST-URS**	**F-URS**	***P***
Cases	148	61	87	-
Sex (M/F)	103/45	45/16	58/29	-
Age (years)	48.4 ± 12.8	48.3 ± 12.7	48.5 ± 13.0	0.917^t^
BMI	24.4 ± 3.4	24.7 ± 2.7	24.1 ± 3.8	0.262^t^
Stone size (cm)	1.47 ± 0.42	1.50 ± 0.45	1.45 ± 0.40	0.530^u^
Stone CT numerical value (Hu)	1302.0 ± 184.6	1288.8 ± 179.0	1311.3 ± 188.9	0.466^t^
Hydronephrosis (cm)	2.9 ± 1.2	2.9 ± 1.3	2.8 ± 1.2	0.739^t^
Preoperative white blood cell count (× 10^9^/L)	6.71 ± 1.73	6.75 ± 1.75	6.69 ± 1.73	0.835^t^
Preoperative bloodneutrophil ratio (%)	59.0 ± 8.4	59.5 ± 7.9	58.6 ± 8.8	0.495^t^
Operative time (min)	45.58 ± 24.03	40.10 ± 20.01	49.43 ± 25.92	0.010^t^
Operative blood loss (ml)	4.35 ± 7.65	4.92 ± 9.51	3.95 ± 6.04	0.364^u^
Success rate ofUAS placement	96.62% (143/148)	96.72%(59/61)	96.55% (84/87)	1.000^χ^
SFR of 1 dayafter operation	39.86% (59/148)	60.66%(37/61)	25.29% (22/87)	< 0.0001^χ^
SFR of 1 monthafter operation	78.38% (116/148)	81.97%(50/61)	75.86% (66/87)	0.375^χ^
Hospitalization cost(yuan)	30,387 ± 7,502	27,686 ± 9,120	32,281 ± 5,420	< 0.0001^t^
Total complication rate	9.46% (14/148)	3.28%(2/61)	13.79%(12/87)	0.031^χ^

*ST-URS, negative pressure combined ureteroscopy (Sotn-ureteroscopy); F-URS, flexible ureteroscopy; BMI, Body Mass Index; UAS, ureteral access sheath; SFR, stone-free rate; P < 0.05 as statistically significant;*

t *Using the Student's t test*;

χ *Using the Chi-squared test*;

u *Using the Mann-Whitney U-test*.

When the preoperative hydronephrosis range ≥ 3.0 cm, compared with the F-URS group, the operation time was shorter (40.38 vs. 52.24 min, *P* = 0.025) and the SFR of 1 day after the surgery was higher in the ST-URS group (66.67 vs. 34.78%, *P* = 0.002, Table 6). The hospitalization cost analysis of these two groups found that ST-URS group was significantly less than that of the F-URS group (*P* < 0.0001). In addition, the incidence of surgical complications in ST-URS group was lower than F-URS group (4.17 vs 13.04%, *P* = 0.241). However, there were no significant differences between the two groups in the operative blood loss, the SFR of 1 month after surgery, and the incidence of surgical complications (*P* > 0.05, Table 6).

**Table 6:** Comparison of Surgical effect in the two groups while the preoperative hydronephrosis range ≥ 3.0 cm.

**Variables**	**Total**	**ST-URS**	**F-URS**	***P***
Cases	94	48	46	-
Sex (M/F)	73/21	37/11	36/10	-
Age (years)	48.5 ± 13.3	48.9 ± 13.7	48.1 ± 13.0	0.770^t^
BMI	25.2 ± 3.3	25.1 ± 3.1	25.3 ± 3.5	0.865^t^
Stone size (cm)	1.44 ± 0.47	1.44 ± 0.52	1.44 ± 0.41	0.942^t^
Stone CT numerical value (Hu)	1086.3 ± 329.8	1073.7 ± 352.4	1099.5 ± 307.8	0.707^t^
Hydronephrosis (cm)	4.1 ± 1.0	4.1 ± 1.0	4.0 ± 1.0	0.578^u^
Operative time (min)	46.18 ± 25.98	40.38 ± 22.97	52.24 ± 27.76	0.025^u^
Operative blood loss (ml)	4.77 ± 8.77	4.44 ± 10.58	5.11 ± 6.46	0.114^u^
SFR of 1 dayafter operation	51.06% (48/94)	66.67%(32/48)	34.78% (16/46)	0.002^χ^
SFR of 1 monthafter operation	79.79% (75/94)	85.42%(41/48)	73.91% (34/46)	0.165^χ^
Hospitalization cost(yuan)	29,297 ± 5,468	26,819 ± 4,180	31,881 ± 5,492	< 0.0001^t^
Total complication rate	8.51% (8/94)	4.17%(2/48)	13.04%(6/46)	0.241^χ^

*ST-URS, negative pressure combined ureteroscopy (Sotn-ureteroscopy); F-URS, flexible ureteroscopy; BMI, Body Mass Index; SFR, stone-free rate; P < 0.05 as statistically significant*;

t *Using the Student's t test*;

χ *Using the Chi-squared test*;

u *Using the Mann-Whitney U-test*.

## Discussion

Ureteral calculi frequently cause renal colic and lead to obstructive urinary tract disease without treatment. Given the development of natural endoscopic instruments and techniques, URS is considered one of the most important methods for the primary treatment of > 10 mm proximal ureteral stones (6). Rigid ureteroscopy is considered to be a preferred operation method for the treatment of the middle and lower ureteral stones (19), but it may be ineffective for treating upper large ureteral stones (19–21). F-URS has excellent SFRs in treating patients harboring proximal ureteral stones smaller than 2 cm (22). Despite the increasing popularity of F-URS, the management of high intrarenal pressure during F-URS has been a clinical dilemma because of its difficulty. While the renal pelvic pressure is high, this may cause the high probability of absorption of liquid, bacteria, and endotoxin into the blood resulting in short-term complications such as SIRS, sepsis, and long-term complication of renal function impairment (23, 24). However, decreasing the perfusion flow to avoid high intrarenal pressure will directly affect the surgical visualization and result in low lithotripsy efficacy. For reducing the renal pelvic pressure, there are many methods such as adding isoproterenol to the surgical perfusion solution using a dual-channel continuous-flow URS and a traditional UAS for F-URS (25–28). In addition, studies have shown that vacuum suctioning can reduce renal pelvic pressure efficiently and significantly increase the safety and efficacy of minimally invasive suctioning PCNL (29–31). Consistent with this, another study showed that a ureteroscopy featuring a vacuum suction system is effective and safe for treating upper urinary tract calculi (32). Despite the acceptance of ureteroscopy with vacuum suction system in urological clinical practice; however, robust comparative data comparing ureteroscopy with suction system and F-URS are lacking. Therefore, we conducted a retrospective study to explore the effects of a novel semirigid ureteroscopy named ST-URS that has an irrigation and vacuum suction platform functioned by its UAS.

Recently, ST-URS, a new lithotripsy operation method in China is widely used in the treatment of ureteral stones. During the operation, the surgeon can adjust the rotary knob to control the negative pressure and actively control the pressure of the suction of stones for simultaneous reduction of the renal pelvic pressure and active suction of the stones (33). Therefore, ST-URS can bring the following surgical effects: 1) at the same time as lithotripsy, the broken stone particles and powder are directly sucked out through the ureteral inlet sheath, thus realizing the integration of crushing and removing stones. 2) By adjusting the pressure of the negative pressure suction valve, the intraoperative pressure in the ureter can be controlled, reducing the possibility of stone escape. 3) The negative pressure suction can suck out the air bubbles, blood clots, and gravel generated during the lithotripsy process so that the surgical vision is clear. 4) The negative pressure suction produces continuous convective water circulation, reducing thermal damage to the ureteral wall caused by the holmium laser. 5) The negative pressure suction can keep the low pressure of the renal pelvis, reducing the risk of infection and bleeding from prolonged surgery and improving surgical visualization (34). In addition, the way of UAS placement is also different between the ST-URS and the flexible ureteroscope. The flexible ureteral sheath is a blind placement method that mainly depends on the experience and feel of the operator or is placed under x-ray fluoroscopy. It may lead to accidental ureteral injury or radiation injury. When the ureter is constricted or twisted, blind placement results in a greater risk of accidental ureteral injury and greater difficulty in operation. Compared with the blind placement method of the flexible ureteral sheath, the rigid UAS of the ST-URS is placed simultaneously under the direct vision and the standard mirror. It is easy for beginners to use and is not easy to damage the ureter, which shortens the learning curve. Therefore, the vision of the whole ST-URS lithotripsy process is clear, and it realizes the integration of crushing and removing stones, which made up for the drawback of ureteroscopy that “only lithotripsy but cannot remove stones at the same time.”

This study compared the clinical efficacy of ST-URS and simple flexible ureteroscope in the treatment of unilateral upper ureteral calculi. Our research suggests that ST-URS has the following advantages in the treatment of upper ureteral calculi: 1) higher SFR of 1 day postoperatively and shorter operative time. In our study, the SFR of 1 day after the operation of the ST-URS group was significantly higher than the F-URS group (63.71 vs. 34.62%, *P* < 0.0001), but the SFR of 1 month after the operation was comparable in the two groups (87.10 vs. 81.41%, *P* = 0.198). Consistent with our results, the study of Zewu Zhu also shows that the suctioning UAS group had a significantly higher SFR of 1 day postoperatively and a significantly shorter operative time in the treatment of renal stones (35). Compared to other studies of patients with similar stone burdens, our SFR result of 1 day postoperatively was superior to that reported in studies in which F-URS was used (36, 37). This is because the negative pressure attraction effect of the ST-URS can suck out larger stones when crushing the stones, reducing the residual stone fragments and stone escape, thus improving the SFR and stone removal efficiency and reducing the operative time. In addition, stone basketing used in the traditional F-URS is time-consuming with incomplete clearance carrying a risk of stone-street formation (38). The use of a suction device had the advantage of removing all stone fragments without requiring a stone basket and thus shortened the operation time. The direct aspiration of small fragments in the ST-URS group would provide better surgical vision and thus lead to higher lithotripsy efficiency. 2) Lower hospitalization costs. In our study, the total hospitalization cost of the ST-URS group was significantly lower than the F-URS group (27,203 vs. 33,220 Yuan, *P* < 0.0001). Compared with the F-URS, ST-URS does not require the insertion of a ureteral stent tube 2 weeks before the operation and does not require the use of a disposable ureteral soft sheath. In addition, the ST-URS reduces the use of flexible ureteroscope and the use of disposables, such as a disposable ureteral soft sheath and the 1.7-Fr basket catheter reducing the medical cost (39). 3) Fewer postoperative complications. In our study, the incidence of infectious complications of the ST-URS group (7.26%) was lower than the F-URS group (10.26%). Zhu et al. also found the incidence of infectious complications was 7.90% in the suctioning UAS group vs. 22.4% in the traditional UAS group and both higher than our results (35). This may be because the average stone size is larger in their study (18.2 and 17.4 vs. 13.7 and 13.5 mm). Both our results suggested the ureteroscopy with a suction device can reduce infectious complications. ST-URS adopts an adjustable negative pressure suction device, the surgeon can actively control the size of the attraction, maintain the low pressure of the renal pelvis, thus significantly decrease perioperative infectious complications (39, 40). Instead of F-URS, the ST-URS is placed under direct vision, which may reduce the damage of the ureter during the insertion process (33). In this study, three patients in the F-URS group had postoperative hematuria while there were no patients with postoperative hematuria or postoperative ureteral injury in the ST-URS group. Consistent with our results, there were no complications of the ureteral mucosa stripping, perforations, and avulsions founded in other studies (33, 39, 40). A study showed that high-power laser lithotripsy settings fired in long bursts with low irrigation flow rates can generate high fluid temperatures in the process of holmium laser lithotripsy (41). In addition, the negative pressure suction produces continuous convective water circulation and higher irrigation flow rates and can take away the heat generated by the holmium laser in time, which may reduce thermal damage to the ureteral wall. Although the incidence of surgical complications in the ST-URS group (7.3%) was lower than the F-URS group (13.5%), but the difference was not statistically significant. This may be related to the small sample size in this study and the results need to be confirmed by a large sample study in the future. Our study combined with these published results showed that the ST-URS with negative pressure suction device has the advantages of high lithotripsy efficacy, fewer complications, more safety, and treating the upper ureteral calculi effectively (33, 35, 39, 40).

Ito et al. reported that the CT value of stones is significantly related to the efficiency of lithotripsy (42). Consistent with their results, we found that when the calculi CT numerical value ≥ 1,000 HU, the operation time of the ST-URS group was shorter than that of the F-URS group (*P* = 0.01) and the incidence of surgical complications of the ST-URS group dramatically decreased than that of the F-URS group (3.28 vs. 13.79%, *P* = 0.031). This reveals that ST-URS also has the advantages of shorter operation time and fewer complications for the treatment of stones with high CT values.

This study has also certain limitations, such as the case number of both the groups in our retrospective study is relatively small. Furthermore, the study was based on the data extracted from a single center. For better validating of the clinical outcomes, we require a multicenter study with a large size sample. Finally, the developed ST-URS cannot achieve real-time monitoring of the actual renal pelvic pressure and should be further improved in the future.

In conclusion, compared with the F-URS, the ST-URS has a shorter operation time, lower hospitalization cost, and higher SFR, especially the SFR of 1 day after the operation. Moreover, the ST-URS has lower postoperative complications in the treatment of ureteral calculi with a CT numerical value ≥ 1,000 HU, so it is a good surgical method for the treatment of upper ureteral calculi.

## Data Availability Statement

The original contributions presented in the study are included in the article/Supplementary Material, further inquiries can be directed to the corresponding authors.

## Ethics Statement

The studies involving human participants were reviewed and approved by Medical Ethics Committee of the First Affiliated Hospital of Xiamen University. The patients/participants provided their written informed consent to participate in this study.

## Author Contributions

BC, HC, and YH designed the study. LL, WZ, FZ, TW, and PB collected the clinical data. ZL, JZ, ZS, BD, HW, and JX analyzed the clinical data. LL and TW wrote and revised the manuscript. All authors approved the final version and agreed to publish the manuscript.

## Funding

This study was funded by the Xiamen science and technology plan (#3502Z20194006), the Young and Middle-aged Backbone Foundation of the Fujian Provincial Health and Family Planning Commission (#2019-ZQNB-26), and the National Science Foundation of China (#81970604 and #82170776).

## Conflict of Interest

The authors declare that the research was conducted in the absence of any commercial or financial relationships that could be construed as a potential conflict of interest.

## Publisher's Note

All claims expressed in this article are solely those of the authors and do not necessarily represent those of their affiliated organizations, or those of the publisher, the editors and the reviewers. Any product that may be evaluated in this article, or claim that may be made by its manufacturer, is not guaranteed or endorsed by the publisher.
